# Prevalence and management of coronary artery anomalies in tetralogy of Fallot at Cheikh Zaid Hospital’s Pediatric Cardiac Surgery Department in Morocco: retrospective study

**DOI:** 10.11604/pamj.2019.34.157.15424

**Published:** 2019-11-22

**Authors:** Mohamed Rida Ajaja, Amine Cheikh, Hicham Rhazali, Mustapha Bouatia, Anas Slaoui, Redouane Abouqal, Amine El Hassani, Younes Cheikhaoui

**Affiliations:** 1 Department of Cardiac Surgery, Cheikh Zaid Hospital, Abulcasis University, Rabat, Morocco; 2 Department of Pharmacy, Cheikh Zaid Hospital, Abulcasis University, Faculty of Pharmacy, Rabat, Morocco; 3 Department of Intensive Care, Cheikh Zaid Hospital, Rabat, Morocco; 4 Pediatrics Hospital, Mohammed V University, Faculty of Medicine and Pharmacy, Rabat, Morocco; 5 Laboratory of Epidemiology and Clinical Research, Mohammed V University, Rabat, Morocco; 6 Department of Pediatrics, Cheikh Zaid Hospital, Mohammed V University, Faculty of Medicine and Pharmacy, Rabat, Morocco

**Keywords:** Tetralogy of Fallot, coronary artery anomalies, congenital cariopathy, cardiac surgery

## Abstract

**Introduction:**

Tetralogy of Fallot (TOF) is one of the most common cyanogenic congenital heart defects. It represents 10% of congenital heart diseases in children. Coronary artery anomalies (CAA) have been reported in 2% to 14% of cases in patients with TOF, according to angiographic, surgical and autopsy series. Many of these anomalies are difficult to detect during surgery. The objective of this article is to study the prevalence of the coronary artery anomalies in patients with TOF as well as their surgical management in our hospital between 2007 and 2015.

**Methods:**

A retrospective study was conducted on 90 patients with TOF aged 1 month to 10 years who were operated on in the Department of Paediatric Cardiac Surgery of Cheikh Zaid Hospital between 2007 and 2015. None of the patients had preoperative coronary angiography and all the anomalies were diagnosed during surgery. Patient clinical data were collected from patient records and from the hospital information system. The qualitative variables are expressed as mean and standard deviation and the quantitative variables are expressed as a percentage. Statistical analyses were performed using SPSS 13.0 software.

**Results:**

Of the 90 patients with TOF followed in the study period, 9 (10%) patients had coronary artery anomalies. We found in 3 (33%) patients an anomalous origin of the left anterior descending coronary artery (LAD) from the right coronary artery (RCA), an anomalous origin of the RCA from the left coronary trunk (LCT) in 1 (11%) patient and a large infundibular branch blocking the pulmonary infundibulum in 5 (56%) patients. All the patients underwent a complete surgical treatment (closure of the ventricular septal defect (VSD) by patch plus stenosis resection plus infundibular enlargement by patch). Eight (89%) patients progressed well in postoperative care and 1 (11%) died immediately after surgery in intensive care.

**Conclusion:**

The coronary anomalies detected in patients with TOF are rare but represent a challenge for the surgical team because of the difficulty of diagnosing them pre-operatively. The management of these anomalies is mainly surgical and the technique used by our team is proved to be safe and effective.

## Introduction

Tetralogy of Fallot is one of the most common cyanogenic congenital heart diseases. It represents 10% of congenital heart disease in children [1]. Coronary artery anomalies (CAA) have been reported in 2% to 14% of patients with TOF according to angiographic, surgical, and autopsy series [2]. Many of these anomalies are difficult to detect during surgery because coronary arteries may be obstructed by adhesions after previous palliative surgery or intramyocardial route and/or epicardial fat [3]. In the total correction of the TOF, the main objective is the ventricular septal defect (VSD) closure and the raising of the obstacle to the ejection of the right ventricle. However, an anomaly of the coronary artery that crosses the right ventricular ejection pathway represents a surgical challenge. Our objective in this work is to evaluate the prevalence of coronary artery anomalies in TOF in children under 10 years old and to present our experience in the surgical management of these anomalies.

## Methods

The study was conducted at the Cheikh Zaid Hospital in Rabat, a hospital containing a paediatric cardiac surgery department that is considered as a reference centre for the management of congenital heart disease. It has a state-of-the-art operating room, a cardiac catheterisation room, 8 post-surgical recovery beds and 2 intensive care units. The service performs an average of 200 surgical procedures annually, of which more than 90% are open heart interventions. A retrospective study was conducted on 90 patients with TOF, aged from 1 month to 10 years, who were operated in the Department of Paediatric Cardiac Surgery of Cheikh Zaid Hospital between 2007 and 2015. None of the patients had a preoperative coronary angiography and all of these anomalies were diagnosed during surgery. Patient clinical data were collected from patient records and from the hospital information system. The qualitative variables are expressed as mean and standard deviation and the quantitative variables are expressed as a percentage. Statistical analyses were performed using the SPSS 13.0 software.

## Results

Of the 90 patients with TOF followed in the study period, 9 (10%) patients had coronary artery anomalies. Among these anomalies, we found in 3 (33%) patients an anomalous origin of the left anterior descending coronary artery (LAD) from the right coronary artery (RCA), an anomalous origin of the RCA from the left coronary trunk (LCT) in 1 (11%) patient, and a large infundibular branch blocking the pulmonary infundibulum in 5 (56%) patients. All patients underwent a complete surgical treatment (closure of the VSD by patch plus stenosis resection plus infundibular enlargement by patch). Eight (89%) patients progressed well in postoperative care and 1 (11%) died immediately after surgery in intensive care. Patient characteristics are presented in the Table 1, Table 2, Table 3.

**Table 1:** Demographic characteristics and clinical symptoms of patients

Patient	Age (months)	Antecedent	Clinical signs
1	120	Blalock Taussing Shunt right and left	Dyspnea, perioral cyanosis
2	7	Repetition Broncho-pneumopathy	Dyspnea, cyanosis, weight loss, growth and developmental delays
3	13	Blalock Taussing Shunt right	Dyspnea, cyanosis
4	3	Nothing to report	Dyspnea, cyanosis
5	9	Broncho-pneumopathy, anoxic malaise	Dyspnea, cyanosis
6	19	Nothing to report	Dyspnea, cyanosis, weight loss, growth and developmental delays
7	1	Nothing to report	Dyspnea, cyanosis, weight loss, growth and developmental delays
8	16	Repetition Broncho-pneumopathy	Dyspnea, cyanosis, weight loss, delay
9	16	Broncho-pneumopathy, anoxic malaise	Dyspnea, cyanosis, weight loss, growth and developmental delays

**Table 2:** Data from the paraclinical examinations

Patient	Electrocardiogram result	Chest radiography result	Transthoracic ultrasound result	Type of anomaly during surgery
1	RSR, RVH	boot shaped heart, pulmonary hypovascularisation, cardiomegaly V2	Normal pulmonary artery branches, Blalock left permeable	Infundibular branch
2	RSR, RVH, RAH	boot shaped heart, cardiomegaly V2	Normal pulmonary artery branches, Blalock left permeable	Infundibular branch
3	RSR, RVH	boot shaped heart, cardiomegaly V2	Normal pulmonary artery branches, small pulmonary ring	Anomalous origin of the LAD from the RCA
4	RSR, RVH	boot shaped heart, pulmonary hypovascularisation, cardiomegaly V2	Normal pulmonary artery branches, normal pulmonary ring	Anomalous origin of the RCA from the LMCA
5	RSR, RVH	boot shaped heart, pulmonary hypovascularisation, cardiomegaly V2	Normal pulmonary artery branches, normal pulmonary ring, dysplastic pulmonary valve	Anomalous origin of the LAD from the RCA
6	RSR, RVH	boot shaped heart, pulmonary hypovascularisation, cardiomegaly V2	Normal pulmonary artery branches, small pulmonary ring	Infundibular branch
7	RSR, RVH	boot shaped heart, pulmonary hypovascularisation, cardiomegaly V2	Normal pulmonary artery branches, small pulmonary ring	Infundibular branch
8	RSR, RVH	boot shaped heart, pulmonary hypovascularisation, cardiomegaly V2	Normal pulmonary artery branches, normal pulmonary ring	Infundibular branch
9	RSR, RVH	boot shaped heart, pulmonary hypovascularisation, cardiomegaly V2	Normal pulmonary artery branches, small pulmonary ring, small left ventricle	Anomalous origin of the LAD from the RCA

**Table 3:** Type of intervention, time of aortic clamping and extracorporeal circulation and duration of stay in intensive care

Patient	Type of intervention	Aortic clamping time/hr:min.	Extracorporeal circulation time/hr:min.	Stay in intensive care/days
1	Infundibular enlargement, intact pulmonary valve ring	1:16	2:09	2
2	Infundibular enlargement, RV-PA conduit	2:23	3:12	2
3	Infundibular and pulmonary arterial enlargement, fused pulmonary valve ring	1:20	2:07	2
4	Infundibular enlargement, intact pulmonary valve ring	1:21	2:08	2
5	Infundibular and pulmonary arterial enlargement, fused pulmonary valve ring	2:26	3:24	4
6	Infundibular and pulmonary arterial enlargement, fused pulmonary valve ring	1:28	2:24	3
7	Infundibular and pulmonary arterial enlargement, fused pulmonary valve ring	1:30	2:12	4
8	Infundibular enlargement, intact pulmonary valve ring	0:46	1:34	2
9	Infundibular and pulmonary arterial enlargement, fused pulmonary valve ring	1:40	2:19	Died

## Discussion

Our series included 90 patients aged 1 month to 10 years admitted to the Department of Paediatric Cardiac Surgery of the Cheikh Zaid Hospital of Rabat for the management of their congenital heart disease. The 90 patients had an irregularly shaped heart with TOF diagnosed by cardiac ultrasound. The treatment for the 90 patients was performed surgically with a heart-lung bypass machine. Between the 90 patients operated, 9 (10%) patients had coronary artery anomalies diagnosed in the surgery. The prevalence found in our series agrees with that reported in the literature. Several studies have reported that coronary artery anomalies (CAA) have been reported from 2% to 14% of patients with TOF according to angiographic series [4-8], surgical series [9-12], and autopsies [13, 14]. The low incidence of coronary artery anomalies in TOF reported by surgical teams can be explained by the fact that many of these anomalies are difficult to detect intraoperatively [4, 9, 10, 15, 16]. In our series, the diagnosis of a coronary artery anomaly was made by the surgical team peroperatively because of the difficulty in detecting them by transthoracic echocardiography, which remains the only means of imaging used by our cardiologists. Surgically, all patients underwent total TOF repair by sternotomy and with a heart-lung bypass machine. Myocardial protection was provided by crystalloid cardioplegia administered at the root of the aorta and repeated every 15 minutes. The total repair of a TOF consists of closure of the VSD by patch, a resection of the pulmonary stenosis by infundibular route, avoiding the coronary artery that crosses the pulmonary pathway. Subsequently, the ejection pathway of the right ventricle is widened by arranging the anomalous coronary artery by patch. Any anomaly of the coronary artery crossing the right ventricle ejection pathway affects the type and timing of complete surgical repair in patients with TOF [17, 18]. Any coronary anomaly that could interfere with right ventriculotomy or resection of the obstruction of the exit pathway should be identified preoperatively because sternotomy may not be the preferred approach for palliative care, and/or because the coronary arteries may be obstructed by adhesions after previous palliative surgery, or by an intramyocardial route [6].

The distribution of the coronary artery is not always definable by surgical observation. The coronary artery may sometimes be hidden by an intramyocardial pathway or by pericardial adhesions, especially after previous surgery [14]. Several techniques have been used to overcome such situations: a right ventricle to pulmonary artery (RV-PA) conduit, a trans-atrial or trans-pulmonary correction, a double ventriculotomy parallel to the anomalous coronary artery, and finally a widening patch placed beneath the anomalous coronary artery branch [19]. All the techniques described above have their drawbacks and are difficult to perform during surgery. In addition, several surgeons suggest total correction of the TOF to prevent ventricular dysfunction and ventricular arrhythmias induced by hypoxia [20, 21]. However, the use of RV-PA conduit in patients with TOF and with coronary artery anomalies makes a second intervention unavoidable, since the ducts have no growth potential and therefore a relative stenosis of the right ventricle ejection pathway will occur [22]. In our series, we adopted the same surgical strategy in 8 cases, consisting of a resection of the pulmonary stenosis by infundibular route after right ventriculotomy while avoiding the coronary barring the right ventricular ejection pathway. In one case, we adopted the Rastelli technique with a RV-PA conduit. The choice of these strategies is justified by the reasons cited above. The enlargement of the lung pathway was done by a synthetic patch, either by passing below the coronary artery (in two cases) or on the edge of the coronary artery after shifting the ventriculotomy. Post-surgical recovery was performed in the same way as other surgically treated cardiovascular anomalies. The occurrence of rhythm disorders or myocardial infarction should be prevented by the reanimation team. In our series, a single patient died in immediate postoperative period because of hemodynamic instability on a hypoplastic left ventricle. The remaining patients have survived and are followed up regularly to the present day.

## Conclusion

The incidence rate of mortality was high and most of the deaths occurred within 12 months after switching to second-line ART. Higher mortality among adult HIV-infected patients was associated with poor adherence, no formal education, not taking IPT, being bedridden at the time of the switch, and not modifying second-line treatment. Improving treatment adherence of patients by providing consistent adherence counseling, providing INH prophylaxis and monitoring patient’s regimen more closely during the first twelve months after switch could decrease mortality of HIV patients on a second-line regimen.

### What is known about this topic

- Coronary abnormalities are difficult to diagnose by the surgical team. The use of angiography or coro-scann allows a better diagnosis when suspicion of the coronary anomalies;
- The surgical team faces operational difficulties in case of discovery of coronary abnormalities intra-operatively;
- A complete correction using an enlargement patch can give good clinical results.

### What this study adds

- Trans-thoracic ultrasound remains the gold standard for resource-limited countries;
- The prevalence of these anomalies is 10% which is consistent with the world literature;
- Complete correction is the most appropriate technique to avoid palliative surgery.

## Competing interests

The authors declare no competing interests.
